# Group differences in feeding and diet composition of wild western gorillas

**DOI:** 10.1038/s41598-022-13728-7

**Published:** 2022-06-10

**Authors:** Terence Fuh, Angelique Todd, Anna Feistner, Giuseppe Donati, Shelly Masi

**Affiliations:** 1 Departement of Social Sciences, Oxford Brookes University, Oxford, UK; 2 Dzanga-Sangha Protected Areas, Bangui, Central African Republic; 3 Unité Eco-Anthropologie, Muséum National d’Histoire Naturelle, CNRS, Université Paris Diderot, 17 place du Trocadéro, 75016 Paris, France; 4 Present Address: WWF Central African Republic Country Programme Office, B.P. 1053 Bangui, Central African Republic; 5 Present Address: Gabon Biodiversity Program, Center for Conservation and Sustainability, Smithsonian Conservation Biology Institute, Gamba, Gabon; 6 Present Address: Fauna & Flora International, Cambridge, UK

**Keywords:** Behavioural ecology, Behavioural ecology

## Abstract

The ecological-constraints model posits that living in larger groups is associated to higher travel costs and reduced nutritional intake due to within-group feeding competition setting upper group size limits. While this is critical for frugivorous mammals, the model is less ubiquitous for folivores who feed on more abundant and evenly distributed food. The seasonally frugivorous diet of western gorillas (*Gorilla gorilla*) provides the opportunity to study the ecological-constraints model in the largest primate species. We investigated how two groups of western gorillas of differing sizes (N = 9, N = 15) in Central African Republic, responded to seasonal variation in fruit availability in terms of activity and diet. We used continuous focal animal sampling during periods of high (July–August 2011) and low (October 2011–January 2012) fruit availability, measured by monthly phenological scores. While diet diversity, resting and moving time did not differ between groups, overall the smaller group spent more time feeding than the larger group although this became less evident when fruit was more available. The smaller group was more frugivorous than the larger group. However, the larger group increased more steeply fruit consumption when fruit was more available, and incorporated more insects, young leaves and bark when fruit was less available, when compared to the smaller group. Up to a certain limit, the flexibility of large, seasonal frugivores to survive on a more folivorous diet may buffer the upper limit group size, suggesting deviation from the ecological-constraints model as in some folivores.

## Introduction

Rainforests are characterized by seasonal and inter-annual variation in the availability of vegetative and reproductive plant parts (e.g.^1–4^). As a result, primary consumers face periods of preferred food abundance and food scarcity (e.g.^5–9^). Variation in food availability is thus expected to affect foraging habits and diet composition, in turn influencing also species’ dietary diversity (e.g.^10,11^). Consequently, different animal species have developed various strategies to optimize their energy budgets^12,13^. Many birds and ungulates migrate seasonally (e.g. birds:^14,15^; ungulates:^16,17^). Other species switch dietary choices to different food categories or to resources that are available year-round (e.g. birds:^18^; antelopes:^19^; monkeys:^2,20^; elephants:^21^; western gorillas:^22–25^). They may vary the daily range and travelling time to optimize foraging effort (e.g. ungulates:^19,26,27^; primates:^28–32^). Primate species respond in different ways to such socio-ecological constraints. Most great apes show clear dietary variation and flexibility in response to seasonal fruit availability (e.g.^25,33–41^).

For species living in stable and cohesive social units, within-group feeding competition may limit foraging efficiency depending on food patch size^42–44^, especially in large groups^45^. Limiting group size (reducing cohesion/forming sub-groups) may be one strategy to cope with reduced food availability^43,46,47^. According to the ecological-constraints model^43,45,48–51^, many primate species alter diet, activity budgets and/or reduce group cohesion in order to stay successfully in large, stable groups. The ecological-constraints model posits that living in larger groups is associated to higher travel costs and reduced nutritional intake due to within-group feeding competition setting upper group size limits. This pattern is clear in most frugivorous primates, but the prediction power of the ecological constraints model is weak for most of folivorous primates living in small groups (folivore paradox:^43,51,52^). This is because food resources of folivores are generally more abundant and widely distributed^53,54^, enabling them to compensate for the increased nutritional requirements by foraging in non-rapidly depleted food patches. However, some folivores support the ecological constraints model by showing high scramble competition within large groups (i.e. the consumption of food by a large number of individuals reduces the amount of food available to the group members^55–59^). Infanticide as a male reproductive strategy in some social mammals^60^ has been suggested as a possible factor explaining the lack of this relationship in other folivores, due to the high number of females with dependent infants and high influx of immigrating and extra-group males, a possible pressure limiting larger group size^61,62^. Similarly, predation risk, which may be higher for terrestrial and smaller bodied species, may also limit group size by constraining group spread to an upper limit^51^. Generally, large groups have greater group spread (e.g.^63^).

Gorillas are the most terrestrial of the great apes and also the largest living primates^64^. They are expected to deviate from the ecological-constraints model as, predicted by their body size, predation risk for adults is low but group cohesion is necessary to limit infanticide risk from extra-group males^65–67^. Western gorilla (*Gorilla gorilla*)’s ranging patterns, activity budgets and even their health and microbiome are strongly affected by seasonal changes in the environment^24,68–75^. As fruit availability increases (and so fruit consumption), western gorillas increase their traveling time and daily path length to locate fruit trees (western gorillas: e.g.^23,24,68,70–72,76,77^). In this framework, western gorillas are particularly interesting as they show a flexible diet and respond to seasonal variation in fruit availability by dramatically modifying their diet from mainly folivorous (> 70% of leaves) to mainly frugivorous (> 70% of fruit; e.g.^22–25,66,76,78,79^). On the other extreme, the Virunga population of mountain gorillas (*G. beringei beringei*) are mainly herbivorous^80^ and have similar average group size to western gorillas (mountain gorillas 10–15 individuals^81,82^; western gorillas 8–9 individuals^83^). However, the maximum observed group size is double in mountain gorillas compared to western gorillas (65 versus 35;^84^ and^85^), raising the question of whether seasonal frugivory may set an upper limit for group size in western gorillas. The diet flexibility of western gorillas thus provides us with the opportunity to increase our understanding of the ecological-constraints model for large species that are not strictly frugivorous or folivorous.

How (temporal) seasonal variation in food availability affects group size and composition of Critically Endangered western gorillas has yet to be investigated. Theoretically, during periods of fruit abundance, western gorillas may face two opposing pressures: the urge to travel and spread out in search of fruiting trees and reduce within-group feeding competition, and the need to maintain adequate group cohesion to avoid infanticide and predation risk (higher on isolated individuals). Thus, the need to seek a fine-tuned balance between these two pressures may limit group size in this seasonal frugivorous species.

Here, we investigate the influence of group size on activity budget and diet of two groups of wild western gorillas, in terms of their response to seasonal variation in fruit availability. We predict that to minimise within-group feeding competition, the larger group will be a) less frugivorous than the smaller group, b) it will increase its travel time to locate either more or larger fruiting trees to meet its energy requirements [e.g. 45, 50]. If the larger group has a less frugivorous diet in comparison to the smaller group, we predict c) it will compensate by increasing feeding time and diet diversity in order to meet energy requirements [e.g. ^45,50^].

## Methods

The study was carried out in Bai Hokou (20° 50' N, 16° 28' E) and Mongambe (2°55'N, 16°23'E) in the Dzanga-Ndoki National Park of the Dzanga-Sangha Protected Areas (DSPA) in south-western Central African Republic (CAR). The 4579 km^2^ DSPA is managed by the CAR government, with significant financial and technical support from WWF. The climate is characterized by a dry season of three months (December-February) and a long rainy season, usually interrupted by a drier period in June-July (DSPA long-term data). In 2011, mean annual rainfall at Bai Hokou was 1200 mm with temperatures varying little over the year, averaging 26.3 °C (mean monthly minimum 19.8 °C and maximum 28.7 °C; long-term data from DSPA). Data were collected by TFN over six months, from July to August 2011 and October 2011 to January 2012, on two different-sized study groups of habituated western gorillas: a smaller group, CAR1, studied in Bai Hokou (N = 9 individuals) and a larger group, CAR2 ranging around Mongambe site (N = 15; see Table 1 for group composition; age/sex classes defined following Breuer et al.^86^). Despite the difference in size, the two groups were comparable since both silverbacks were estimated of comparable age, thus the age of the group (both including infants and juveniles). The two groups ranged 10 km apart and home ranges did not overlap (long-term data from DSPA). It was not possible to record data blind because our study involved wild focal animals in the field.

**Table 1:** Total hours of focal animal sampling per each group member of the two study groups.

Age/sex category	Group	Total hours of focal
Silverback	Small	25.1
Adult female 1	Small	7.3
Adult female 2	Small	22
Blackback	Small	17.7
Juvenile 1	Small	21.1
Juvenile 2	Small	19.7
Juvenile 3	Small	21.3
Juvenile 4	Small	20.4
Silverback	Large	22.6
Adult female 1	Large	18.8
Adult female 2	Large	18.9
Subadult	Large	13.4
Juvenile 1	Large	10.3
Juvenile 2	Large	17.8

Age/sex categories are as defined by^86^ (Juvenile: 4–7.5, Subadult: 7.5–11, Blackback: 11–14, Young Silverback: 14–18, Adult Female: > 10 yr and Silverback: > 18 yr). Real group compositions a) Small group (CAR1): one silverback, two adult females, one blackback, four juveniles and one infant, and b) Large group (CAR2): one silverback, four adult females, three sub-adults, four juveniles and three infants.

### Behavioural data

Data on gorilla activities were collected during half-days (from 6:30 am to c.a. 12:00 pm or from 12:00 pm to 4:30 pm) via continuous focal animal sampling^87^, rotating the focal individuals to balance sample size. Focal animal sampling was carried out eight or nine half-days per month on group CAR1 (N_tot_ = 54 days) and seven half-days per month on group CAR2 (N_tot_ = 42 days). For group CAR1, all group members (n = 9) were focal followed. For logistical reasons (the researcher presence at the site) for group CAR2 , seven out of 15 group members were chosen to represent the whole group across the study period: the silverback, two adult females, one subadult male, two juveniles and one infant. Therefore, across the study period each individual was sampled at least once per month ensuring even sampling between morning and afternoon for each individual (see Table 1 for sample sizes per age/sex class and group). Since infants did not follow the adult diet, we excluded them from all analyses.

During focal sampling, we continuously recorded the position of the focal individual (on ground or tree) and its activity classified into four main categories (modified from^24^ and^88^): feeding (including foraging and food processing), moving (walking, running, climbing up or down trees), resting (stationary, sitting or lying down, autogrooming), social (playing, interacting with, vocalizing or displaying towards another individual), and other (vigilance, nesting, drinking, etc.). We also noted the food species and the food type consumed by the gorillas, i.e. fruit, young leaves, all leaves (mature and young leaves), stems (terrestrial herbaceous vegetation, aquatic herbs, etc.), barks, insects, and others (coprophagy, dry leaves, etc.).

### Statistical analysis

For all focal animals (excluding infants), the average daily and average seasonal percentages of time spent feeding, moving, resting and socializing were calculated. The half-day proportion of feeding time spent on each food category (fruits, all leaves, young leaves, stems, insects, barks) was also calculated. Food diversity was calculated as the half-daily number of food species (plant, insect and fungus) consumed per focal animal.

To analyse differences in proportions of activity budget and diet composition between the two groups and its interaction with fruit availability, we used Generalized Linear Mixed Models (GLMM) with a binomial error distribution and logit link function^89^. Fruit availability (included as monthly phenological score) and groups were used as fixed factors while individuals were added as a random effect. We built a model for the following response variables: the time spent feeding, moving, resting, social, feeding on fruit, all leaves (mature and young leaves from the same species), young leaves (only), stems and insects. We kept separated all leaves and young leaves, since gorillas eat mainly young leaves-only on trees which are scattered and thus more difficult to find than the widely distributed shrubs, from which all leaves are eaten on the ground. To model diet diversity (response variable) we used a GLMM with Poisson error distribution and logit link function to account for count data^89^. Fixed and random factors were the same as for the previous models. In all models we included the interaction of the groups with monthly fruit availability calculated as the monthly phenological score. The Variance Inflation Rate (VIF) was calculated for all predictors to control for multicollinearity issues. All statistical analyses were run in SPSS 27.

### Compliance with ethical standards

This research adhered to ethics and healthy protocols and legal requirements of the governments of Central African Republic. All applicable international, national and/or institutional guidelines for the care and use of animals were followed, including adherence with the ARRIVE guidelines.

## Results

### Activity budget

**Figure 1:**
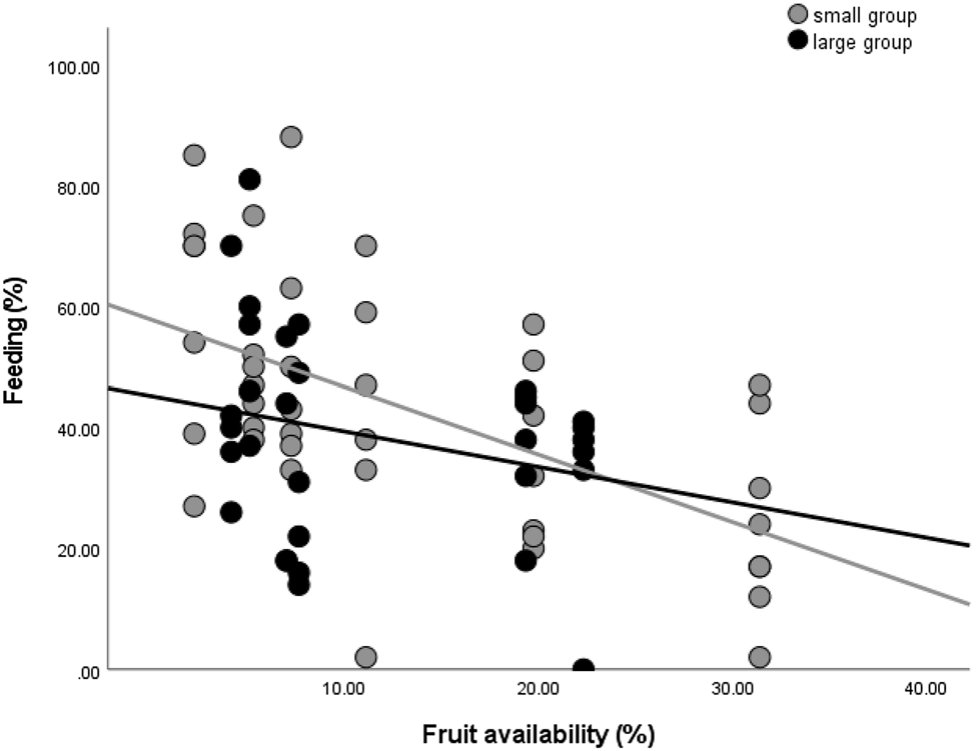
Group comparison for percentages of time spent feeding in relation to fruit availability. Fruit availability is measured as monthly phenological score (see “Methods”). Each dot indicates the average monthly percentage of time spent feeding for each focal individual. The lines are best-fit-lines for each group data points.

The smaller group spent generally more time *feeding* than the larger group (the larger group being always the reference: b = 0.477, stand. error = 0.205, t = 2.333, *P* = 0.023). Overall, feeding time decreased with the increase in fruit availability (b = − 0.028, stand. error = 0.005, t = − 5.452, *P* < 0.001). Groups behaved differently in relation to changes in fruit availability, feeding time dropped more steeply in the smaller group compared to the larger group with the increase in fruit availability (b = − 0.021, stand. error = 0.006, t = − 3.491, *P* = 0.001; Fig. 1).

The groups did not differ in the time spent *moving* (b = − 0.76, stand. error = 0.191, t = − 0.396, *P* = 0.693) but overall they moved more when fruit availability was higher (b = 0.024, stand. error = 0.006, t = 3.897, *P* << 0.001). There was no difference between the two groups in the moving response when fruit availability changed (b = − 0.003, stand. error = 0.007, t = − 0.460, *P* = 0.647).

No significant effect was found for *resting* time neither between groups (b = − 0.236, stand. error = 0.315, t = − 0.749, *P* = 0.456), nor in relation to fruit availability (b = 0.004, stand. error = 0.005, t = 0.817, *P* = 0.416) or their interaction (b = 0.005, stand. error = 0.006, t = 0.870, *P* = 0.387).

No differences were found between the groups in time spent in *social activities* (b = − 0.103, stand. error = 0.7190, t = − 0.143, *P* = 0.887). However, social activities increased with higher fruit availability (b = 0.050, stand. error = 0.013, t = 3.770, *P* < 0.001) and this was more evident for the smaller group when compared to the larger group (b = 0.033 stand. error = 0.015, t = 2.221, *P* = 0.030).

### Diet diversity and composition

*Diet diversity* did not significantly correlate with fruit availability (b = 0.006, stand. error = 0.008, t = 0.710, *P* = 0.480), nor was there an overall difference between the two groups (b = − 0.074, stand. error = 0.203, t = − 0.367, *P* = 0.715) or a difference response of the groups depending on fruit availability (b = − 0.005, stand. error = 0.010, t = − 0.552, *P* = 0.582).

**Figure 2:**
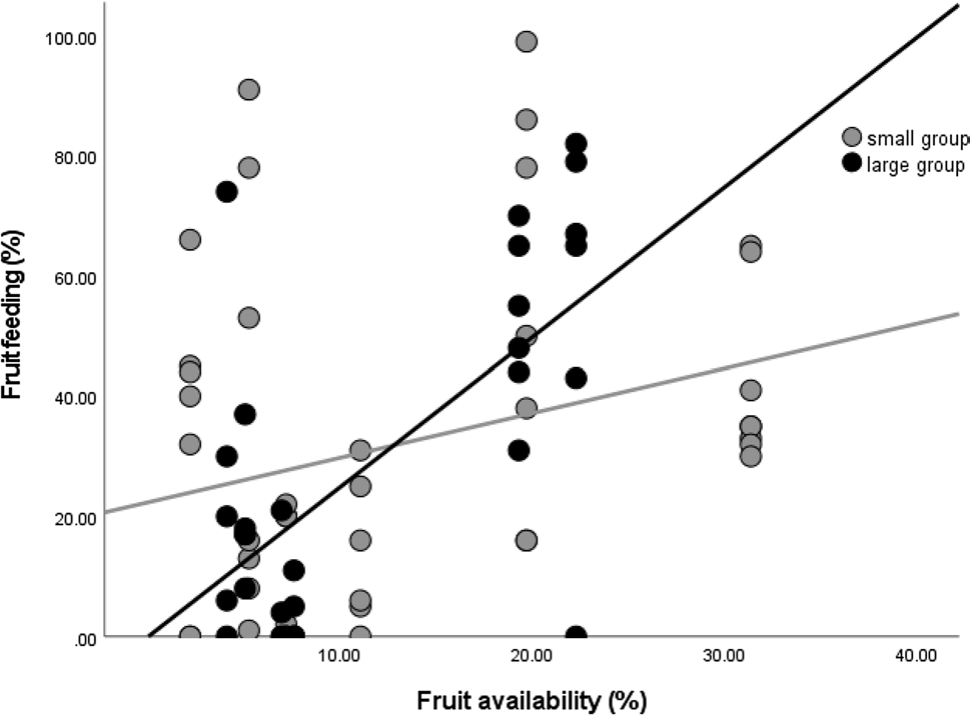
Group comparison for percentages of time spent feeding on fruit in relation to fruit availability. Fruit availability is measured as monthly phenological score (see “Methods”). Each dot indicates the average monthly percentage of time spent feeding on fruit for each focal individual. The lines are best-fit-lines for each group data points.

*Fruit* consumption was overall higher in the smaller group than in the larger group (b = 1.371, stand. error = 0.301, t = 4.548, *P* < 0.001). As expected, feeding on fruit increased with fruit availability (b = 0.127, stand. error = 0.006, t = 21.357, *P* < 0.001) and this was more evident for the larger group than the smaller group (b = − 0.094, stand. error = 0.007, t = − 13.846, *P* << 0.001; Fig. 2).

The groups did not differ in the consumption of *all leaves* (young and mature leaves from the same species; b = 0.717, stand. error = 0.526, t = 1.362, *P* = 0.178). Overall, gorillas decreased all leaf consumption when fruit was more available (b = − 0.069, stand. error = 0.008, t = − 8.992, *P* << 0.001). However, there was no difference in the group response when fruit availability varied (b = − 0.010, stand. error = 0.009, t = − 1.064, *P* = 0.291).

Similarly, *young leaves’* consumption also did not differ between the two groups (b = − 1.916, stand. error = 1.847, t = − 1.037, *P* = 0.303) and it was negatively correlated with fruit availability (b = − 0.148, stand. error = 0.018, t = − 8.196, *P* << 0.001). However, this correlation was significantly weaker in the smaller group than in the larger group (b = 0.107, stand. error = 0.022, t = 4.727, *P* << 0.001). In other words, the larger group increased young leaves consumption more strongly when fruit availability decreased.

*Stem* consumption did not differ between the study groups (b = − 0.130, stand. error = 0.311, t = − 0.418, *P* = 0.677) nor in relation to fruit availability as main effect (b = 0.005, stand. error = 0.0059, t = 0.826, *P* = 0.412). However, when fruit availability increased the smaller group increased stem consumption more significantly in comparison to the larger group (b = 0.016, stand. Error = 0.007, t = 2.296, *P* = 0.025).

**Figure 3:**
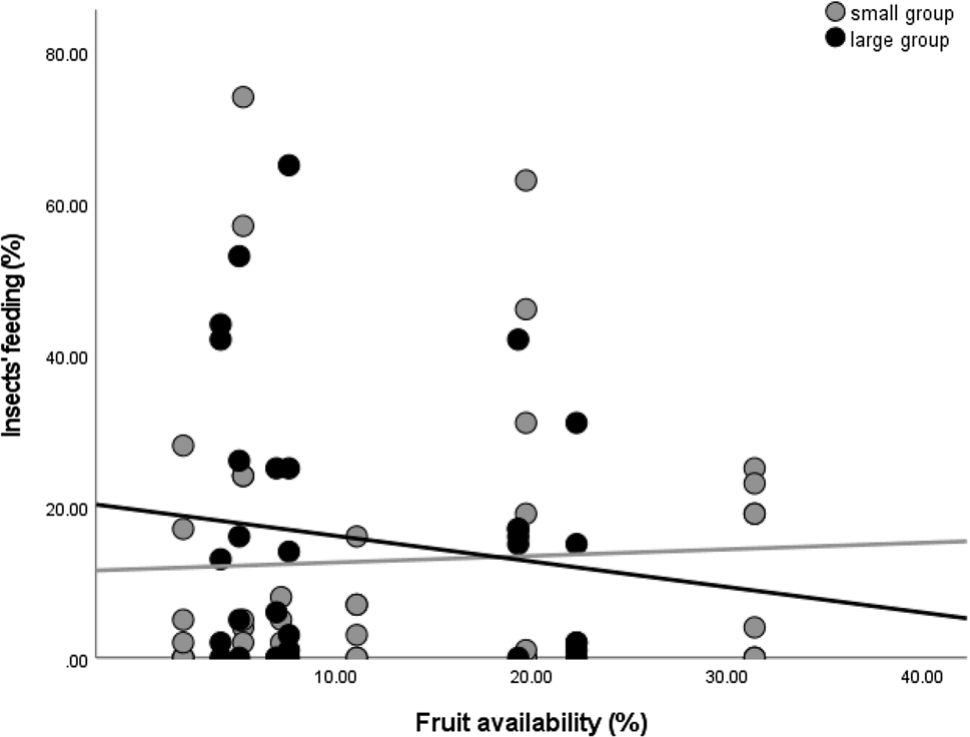
Group comparison for percentages of time spent feeding on insect in relation to fruit availability. Fruit availability is measured as monthly phenological score (see “Methods”). Each dot indicates the average monthly percentage of time spent feeding on insect for each focal individual. The lines are best-fit-lines for each group data points.

*Insect* consumption was overall lower in the smaller group than in the larger group (b = − 0.770, stand. error = 0.365, t = − 2.108, *P* = 0.039). It generally decreased with higher fruit availability (b = − 0.028, stand. error = 0.007, t = − 4.036, *P* < 0.001) and this was less evident for the smaller group than the larger group (b = 0.036, stand. error = 0.008, t = 4.248, *P* < 0.001). In other words, the larger group increased more strongly insect consumption when fruit was less available (Fig. 3).

Groups did not differ in *bark* consumption (b = − 2.249, stand. error = 1.599, t = − 1.406, *P* = 0.164). Gorillas decreased bark consumption when fruit availability increased (b = − 0.444, stand. error = 0.065, t = − 6.792, *P* < 0.001) but this was less evident in smaller than the larger group (b = 0.280, stand. error = 0.070, t = 3.958, *P* < 0.001). Thus, the larger group increased bark consumption more when fruit was less available.

## Discussion

Generally, diet and feeding time were more affected by group size than the rest of the activity budget. Resting time and diet diversity were rather independent of the variation in both fruit availability and/or group size. Time spent moving and feeding on all leaves (young and mature leaves) increased with fruit availability, but they were rather independent of group size. All other variables were affected by both fruit availability and group size simultaneously. Particularly, when fruit availability increased in the forest, the larger group decreased feeding time and increased fruit consumption more steeply when compared to the smaller group. When fruit availability decreased, the larger group increased more strongly consumption of young leaves, stems, insects and barks when compared to the smaller group. Even though we could compare only two rare groups of habituated western gorillas, these differences can possibly be linked to the different group size. Western gorillas are seasonal frugivores (e.g.^22–25,79^) and our study showed that group size may influence their diet composition, feeding and social time, in response to seasonal variation in fruit availability.

As consequence of increased group size, many animals consequentially increase feeding time (e.g. ungulates:^90,91^; primates:^42,51,92^). Like many primates (e.g.^93^), western gorillas' feeding time is greatly influenced by seasonal availability of fruit. In this regard, our results corroborate previous findings of lower feeding time during the high frugivory periods (correlated with fruit availability, this study) because of the higher energetic content of fruit (e.g.^24,73^). However, the two groups responded differently in relation to changes in fruit availability. When fruit availability increased, feeding time dropped less steeply in the larger group compared to the smaller group likely because of the expected increased within-group competition resulting from a larger group size. Similarly, the larger group increased more steeply fruit consumption than the smaller group when fruit was more available. This “energy maximiser” strategy is rather used by primates inhabiting unpredictable seasonal environments, which increase feeding time when fruit is more available, exceeding daily caloric requirements in order to store fat to subsist on lower quality food during lean seasons (e.g.^35,94–96^). It is likely that the increased feeding time of the larger group when fruit is more available is in response to the higher energy requirements demanded by a larger group size^97^.

As expected, the smaller group was also generally more frugivorous than the larger group. In addition, when fruit availability was lower the individuals of the larger group significantly increased insect, young leaves and bark consumption when compared to the smaller group. These findings suggest that group size may be a limiting factor for frugivory, particularly when fruit is scarce. The diet differences between the groups are unlikely to be the result of differing availability of fruit within the home-ranges of the study groups, since the phenological comparison showed no major differences in the number of trees fruiting or the fruit score (see Supplementary Materials).

In response to a more folivorous diet, the dietary diversity of a species can widen^98^. Contrary to our predictions, the higher folivory/insectivory in the larger group when fruit was less available was not associated with a broader diversity of food species ingested when compared to the smaller group. Western gorillas already possess a broad diet^25,78^, extending it further is probably not a profitable feeding strategy for them.

The larger group compensated the higher nutritional needs by changing the proportion of time spent feeding on other foods. One strategy is to incorporate lower quality food in the diet (as opposed to fruit) in order to meet the nutritional requirements of a larger group (ungulates:^26,99^; primates:^12,100^). Leaf, young leaf and bark consumption by the gorillas were generally higher when fruit was less available in the forest, serving likely more as fallback food as observed in other primates [e.g.^101^]. When compared to the smaller group, the seasonal variation of young leaf and bark consumption of the larger group was higher. Since non-reproductive plant parts are generally less patchily distributed than fruit (e.g.^102^; but see^103^), their inclusion allows likely larger groups to maintain higher group cohesion, and potentially decrease infanticide and predation risks^65,66^. Being hind-gut fermenters (thanks to their large body size) like other large mammals, gorillas can extract from a folivorous diet a large quantity and diversity of macro- and micro-nutrients^25,104–106^. However, consumption of all leaves, mainly from shrubs, did not vary between groups and in relation to fruit availability, likely because they are always available and less nutritious than young leaves or insects.

Another strategy of the larger group was to increase more insect consumption when fruit was scarce, when compared to the smaller group. Since the two study sites are only 10 km a part each other as a part of the same continuous forest, we do not expect this difference (occurring only when fruit availability was low) being the result of a different termite availability between the sites. Insects are highly nutritional food resources (e.g.^107,108^), allowing many primate species to survive in seasonal environments (e.g.^108–110^). Our result contrasts with several studies showing increased insect exoskeleton remains in western gorilla faeces during the rainy (high fruit availability) season when termite mounds maybe easier to break/open (e.g.^71,76,111^). However, for larger groups, increasing insectivory while decreasing frugivory during the (dry) low fruit availability may be more profitable for maintaining group cohesion, since termite mounds are less dispersed than the few individual fruiting trees available (Masi and Todd, unpublished data).

Social mammals like primates and carnivores are expected to respond to increased group size by increasing travel time (e.g.^42,43,49,112^). Contrary to our predictions, group size did not affect the time spent moving in western gorillas (neither as main effect nor in association with fruit availability), contrary to what has been found for other primates^113^. In fact, western gorillas already incur additional costs for longer daily travel distance and time when feeding on fruit (e.g. this study;^23,24,69,71^). These higher energetic costs are compensated by the high energetic content of fruit confirming the tendency of a previous study^24,25^. When feeding on fruit, they also likely increase group spread (within limits;^24,114^). The group spread may be more evident in the larger group, since decreased social cohesion and reduced synchronisation of animal activities may be associated to increased group size (e.g. ungulates:^90,114^). This is corroborated by the higher social activities found in the smaller group during the high fruit availability period, allowed by a higher group cohesion among group members, when compared to the larger group.

## Conclusions

Overall, while diet diversity, time spent resting and moving seem to be rather independent of group size, in the western gorillas the response to increased group size, possibly due to higher within-group competition, was a greater flexibility in feeding time and diet composition.

In particular, group size was a limiting factor for frugivory during periods of low fruit availability, when western gorillas are more folivorous. Since the ecological-constraints model shows that within-group feeding competition sets the upper limit of group size for frugivores^45,49–51^ and only for some folivores^56–59^, the flexibility of seasonal frugivores to survive on a more folivorous diet may suggest larger upper group size limit when compared with cohesive strictly frugivorous species. This may explain why frugivorous western gorillas have a similar average group size when compared to the herbivorous mountain gorillas; however, their frugivory explains why in this species the maximum observed group size is lower (although a multi-male vs a single male group strategy also plays a role in this equation;^80–85^). Furthermore, in western gorillas where secondary female dispersal can occur multiple times, larger groups are one of the correlates of adult females transferring to other groups^116–119^. This may be the consequence of heightened infanticide and predation risk in larger groups, where group members need to spread further to feed on fruit^24,114^. Given the dietary flexibility, infanticide is likely a key pressure for limiting group size in this large-bodied seasonal frugivorous species rather than predation (although leopards prey on gorillas;^67^).

Shedding light on the drivers of the evolution of sociality and grouping patterns in our closest relatives, this study contributes to our knowledge on behavioural flexibility in relation to socioecological changes which can be relevant for human evolution.

## Supplementary Information

Supplementary Information 1.
