# Supplementary material for: Group differences in feeding and diet composition of wild western gorillas
Source: Sci Rep. 2022 Jun 10;12:9569. doi: 10.1038/s41598-022-13728-7 (PMC9187766; doi:10.1038/s41598-022-13728-7)
Supplement: Supplementary file 1 [file 41598_2022_13728_MOESM1_ESM.odt]

Group differences in feeding and diet composition of wild western gorillas

Terence Fuh1, 2 Angelique Todd2, Anna Feistner2, Giuseppe Donati1*, Shelly Masi3* 

1Departement of Social Sciences, Oxford Brookes University, Oxford, UK

2Dzanga-Sangha Protected Areas, Central African Republic

3Unité Eco-anthropologie, Muséum National d’Histoire Naturelle, CNRS, Université Paris Diderot, 17 place du Trocadéro 75016 Paris, France

*Equal contribution

Correspondence to: 

Terence Fuh Neba, WWF Central African Republic Country Programme Office, B.P. 1053 Bangui, Central African Republic, [terencefuh@yahoo.com](mailto:terencefuh@yahoo.com) 

Authors’ current affiliation:

TNF: Terence Fuh Neba, WWF Central African Republic Country Programme Office, B.P. 1053 Bangui, Central African Republic

AF: Gabon Biodiversity Program, Center for Conservation and Sustainability, Smithsonian Conservation Biology Institute, Gamba, Gabon

AT: Fauna & Flora International, Cambridge, UK

Supplementary Information

Phenological comparison of the two study sites

As an indicator of habitat food availability [1], the phenology of new leaves, flower and fruit production of important arboreal gorilla foods (occurring for > 1% of gorilla feeding time, following [2]) was monitored monthly along permanent transects within the home range of each group; in Bai Hokou (365 individual trees of 32 species) and in Mongambe (314 trees of 34 species). To assess the availability of young leaves and fruits on individual plants, we used a 0–4 score (0=none, 1=rare, 2=few, 3=common, 4=abundant). Ripe fruits were scored separately from unripe fruits, and both fruits on the ground and on the tree were considered when assessing the score [2].

We used the Wilcoxon test for matched samples to compare phenology of important foods between sites, by comparing average monthly proportions of fruiting and leafing trees and scores weighted to account for sample size. 

During the six months of the study, the percentage of leafing trees in Bai Hokou (median% [min-max%]=37.8% [22.8-44.4%], N=6) did not differ significantly from that in Mongambe (26.4% [14.9-40.9], Wilcoxon test: N=6, Z=-1.352, P=0.176), but the young leaf availability score in Bai Hokou (Mdn [min-max]=0.4 [0.3-0.5], N=6) was significantly lower than in Mongambe (Mdn=0.8 [0.5-1.1], N=6, Z=-2.028, P=0.043). The percentage of fruiting trees in Bai Hokou (9.15% [2.22-31.39%], N=6) did not differ from that in Mongambe (7.30% [4.13-22.30%], N=6, Z=-0.314, P=0.753), nor the fruit availability score (Bai Hokou: Mdn [min-max]=0.4 [0.2-0.7], N=6; Mongambe: Mdn=0.5 [0.2-1.2], N=6, Z=-0.507, P=0.612), indicating that fruit availability did not differ between the home ranges of the two groups.

Cited literature of the Supplementary Information

[1] Chapman CA, White FJ, Wrangham RW (1994) Party size in chimpanzees and bonobos: a reevaluation of theory based on two similarly forested sites. In: Wrangham RW, McGrew WC, de Waal FBM, Heltne PG, editors. Chimpanzee cultures. Cambridge: Harvard University Press p 41–57

[2] Masi S, Mundry R, Ortmann S, Cipolletta C, Boitani L, Robbins MM (2015) The influence of seasonal frugivory on nutrient and energy intake in wild western gorillas. PloS One 10(7)
